# Assessment of brain midline shift using sonography in neurosurgical ICU patients

**DOI:** 10.1186/s13054-014-0676-9

**Published:** 2014-12-09

**Authors:** Julie Motuel, Isaure Biette, Mohamed Srairi, Ségolène Mrozek, Matt M Kurrek, Patrick Chaynes, Christophe Cognard, Olivier Fourcade, Thomas Geeraerts

**Affiliations:** Anesthesiology and Critical Care Department, Equipe d’accueil “Modélisation de l’agression tissulaire et nociceptive”, University Hospital of Toulouse, University Toulouse 3 Paul Sabatier, Toulouse, France; Neuroradiology Department, University Hospital of Toulouse, University Toulouse 3 Paul Sabatier, Toulouse, France; Department of Anesthesia, University of Toronto, Toronto, Canada; Department of Neurosurgery, University Hospital of Toulouse, University Toulouse 3 Paul Sabatier, Toulouse, France

## Abstract

**Introduction:**

Brain midline shift (MLS) is a life-threatening condition that requires urgent diagnosis and treatment. We aimed to validate bedside assessment of MLS with Transcranial Sonography (TCS) in neurosurgical ICU patients by comparing it to CT.

**Methods:**

In this prospective single centre study, patients who underwent a head CT were included and a concomitant TCS performed. TCS MLS was determined by measuring the difference between the distance from skull to the third ventricle on both sides, using a 2 to 4 MHz probe through the temporal window. CT MLS was measured as the difference between the ideal midline and the septum pellucidum. A significant MLS was defined on head CT as >0.5 cm.

**Results:**

A total of 52 neurosurgical ICU patients were included. The MLS (mean ± SD) was 0.32 ± 0.36 cm using TCS and 0.47 ± 0.67 cm using CT. The Pearson’s correlation coefficient (r^2^) between TCS and CT scan was 0.65 (*P* <0.001). The bias was 0.09 cm and the limits of agreements were 1.10 and -0.92 cm. The area under the ROC curve for detecting a significant MLS with TCS was 0.86 (95% CI =0.74 to 0.94), and, using 0.35 cm as a cut-off, the sensitivity was 84.2%, the specificity 84.8% and the positive likelihood ratio was 5.56.

**Conclusions:**

This study suggests that TCS could detect MLS with reasonable accuracy in neurosurgical ICU patients and that it could serve as a bedside tool to facilitate early diagnosis and treatment for patients with a significant intracranial mass effect.

## Introduction

Brain midline shift (MLS) is a life-threatening condition that requires urgent diagnosis and treatment. In addition to the clinical examination, computed tomography (CT) scan has become the corner stone for the care of neurosurgical patients. In 1977, Becker *et al.* noted a two-fold increase in mortality when the MLS exceeded 1 cm (53% versus 25%) [1]. Recently, a MLS above 0.5 cm on the initial brain CT scan has been shown to predict poor neurological outcome with a positive predictive value of 78% [2] whereas only 14% of cases without MLS on CT scan were shown to be associated with a poor outcome [3]. MLS on CT has been found to be correlated with the Glasgow coma score [4-6] and with other CT signs of injury severity (compressed basal cisterns, mass lesions or traumatic sub-arachnoid hemorrhage) [7-11]. A CT scan classification based on data from the Traumatic Coma Data Bank was proposed by Marshall *et al.* [12], including a MLS >0.5 cm as one of the main CT criteria for the severity of traumatic brain injury (TBI) [12,13] and a multivariate analysis of a cohort of over 10,000 TBI patients showed that the compression of the third ventricle and a MLS >0.5 cm were both major predictors of mortality within the first 15 days after injury [14]. Similarly, Ropper observed that, following stroke, alteration of consciousness was directly proportional to the MLS on CT scan [15] and Pullicino *et al*. found that both MLS (*P* = 0.001) and coma (*P* = 0.019) were independent predictors of mortality at 15 days following acute stroke [16]. The mass effect associated with a MLS after ischemic or hemorrhagic stroke is considered one of the major outcome predictors.

The early detection of a MLS in neurosurgical ICU patients is thus very important because it allows the implementation of an appropriate treatment plan (North American recommendations from 2006 call for a surgical evacuation in the case of a MLS >0.5 cm in the presence of severe TBI, extradural, subdural or intracerebral hematoma) [17-19]. However, even though head CT is considered to be the gold standard to diagnose MLS, serial CTs in neurosurgical ICU patients can be associated with significant morbidity related to their transport [20] and their value has, therefore, been questioned [21].

Along with important recent technological advances, sonography of the brain has been shown to be able to visualize most of the intracerebral structures [22]. This technology is non-invasive, associated with low radiation exposure, available at the bedside and has been used as an additional tool for the evaluation of acute ischemic stroke patients. Seidel *et al.* described in 1996 a simple method to determine the MLS with sonography [23]. This seemed to correlate well with findings on CT [24-27] and to be able to serve as an early outcome predictor by rapidly detecting a significant MLS in acute stroke [28,29]. However, this was described only in one small study of patients with TBI [30].

The purpose of our study was to assess the reliability of transcranial sonography (TCS) compared to CT scan to diagnose MLS among various types of neurosurgical ICU patients, including patients after decompressive craniectomy, with subcutaneous hematomas or other conditions that could possibly interfere with this sonographic view. Our secondary objectives were to compare the reproducibility of the TCS measurement between two different ultrasound units and to study the correlation between MLS and intracranial pressure (ICP).

## Methods

This prospective study was conducted between July and October 2010 in a 12-bed neurosurgical ICU of a French University Hospital. All patients with an indication for head CT scan (ordered at the discretion of the attending physician) were eligible for inclusion. The research protocol was approved by the local Research Ethics Board (REB - Comité d’éthique de la recherche, Centre Hospitalier Universitaire de Toulouse, approval 39–1011, including a waiver for the need of a formal written consent form). In accordance with French law (article R1121-3 of the French public health code), a detailed information letter was given to the patients and their relatives including explanations about their right to refuse participation in the study, but no need for a formal signature was required.

### Ultrasound determination of MLS

**Figure 1:**
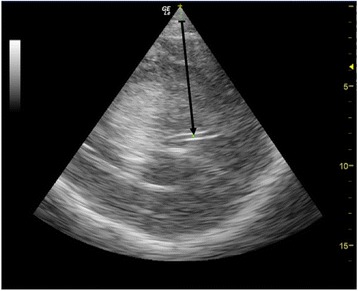
**Example of visualisation of the third ventricle with brain sonography, the arrow pointing to the centre of the third ventricle.**

The ultrasound MLS was measured through the temporal acoustic bone window using a low frequency (2 to 4 MHz) probe as soon as possible before or after the head CT. The third ventricle was identified as a double hyperechogenic image over the midbrain; the distance between the external bone table and the centre of the third ventricle was then measured bilaterally (Figure 1). The difference between two measures (A and B) divided by two was used to calculate the MLS: MLS = (B - A)/2.

By convention, A represented the measure taken on the left side and B on the right side. The resulting values were thus positive if the mass effect was right sided and negative if the mass effect was left sided. The operator (JM) was blinded to the results of CT scans and had previous experience in TCS (but only for Doppler assessment, without MLS measurement).

The measures were taken using the M-Turbo ultrasound system (SonoSite, Bothell, WA, USA). To study the reproducibility of TCS measures between two different machines, the MLS measurement was immediately repeated with a different sonograph (Logiq e, General Electric, Wauwatosa, WI, USA) in one subset of patients.

### Computed tomography scan

The CT scan findings were described using the Marshall score [12,13].

**Figure 2:**
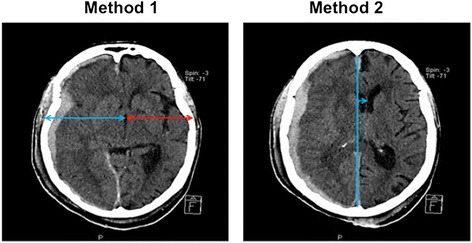
**Example of MLS measure with CT scan: on the left, method 1 (in the same plane as sonography through the third ventricle), and on the right, method 2 (measuring the distance between the ideal median line and the septum pellucidum).** CT, computed tomography; MLS, midline shift.

The measurement of the MLS with CT scan was done twice for each patient (Figure 2):

- The first measurement (method 1) was taken in the same plane as the sonographic measurement: the distance between the external bone table and the centre of the third ventricle was measured from the CT slice among the 5-mm-wide cuts in the orbito-meatal plane that allowed visualising the third ventricle [30].
- The second measurement (method 2), normally used by our neuroradiologists, measured the distance between the ideal mid line and the septum pellucidum [11], where the largest deviation was seen.

These two measurements from the head CT were performed by a neuroradiologist (IB), who was blinded to the ultrasound results and clinical data.

A finding of a MLS >0.5 cm in the CT scan method 2 was considered a significant MLS.

For cases where there was no external bone (that is, after decompressive craniectomy), the distance between duraplasty at the side of the craniectomy and the third ventricle was taken for both CT scan method 1 and TCS.

### Statistical analysis

The correlations between TCS and CT scan measures of the MLS, between MLS and ICP, as well as between the measures from the two sonographs were all analysed by linear regression with Pearson’s correlation coefficient. The agreement between the methods was studied by the Bland and Altman method with calculation of bias and the limits of agreement [31]. Finally, the diagnostic performance of TCS to detect a significant MLS (>0.5 cm calculated with method 2 from the CT scan) was estimated by analysis of the receiver operating characteristic (ROC) curve. Data are expressed as mean (± SD) or median (± range). The agreement between sonography units was assessed by weighted kappa-statistic. Hence, the two ratings were first ‘presence of significant MLS’ (for example, when MLS assessed on TCS was equal to or above a sonographic cut-off extracted from ROC analysis) and second ‘absence of significant MLS’ in the other case. The statistical analysis was carried out using MedCalc® (Mariakerke, Belgium), Statview®, Cary, NC, USA (SAS Institute Inc, USA) and Stata SE® 12.0 (College Station, TX, USA) softwares.

## Results

Fifty-two consecutive adults patients were included (age: 47.1 ± 23.3 years, gender: 73% male, height: 169.8 ± 13.1 cm, weight: 72.5 ± 17.8 kg). The median Simplified Acute Physiology Score (SAPS II) was 41.5 (range 8 to 92). Table 1 summarizes the reasons for admission to the ICU.

**Table 1:** **Reasons for admission to the neurosurgical ICU**

**Reasons for admission**	**Number**	**Percentage**
Severe traumatic brain injury	31	59.6%
Postoperative care	7	13.5%
Severe subarachnoid hemorrhage	8	15.4%
Intracranial hematoma	4	7.7%
Acute ischemic stroke	2	3.8%
Total	52	100%

The hemodynamic and biological parameters at the time of sonography are summarized in Table 2. An ICP probe was in place at the time of sonography in 30 patients. Twelve patients were admitted to the neurosurgical ICU after decompressive craniectomy. Decompressive craniectomy was always a large unilateral fronto-temporo-parietal craniectomy with duraplasty.

**Table 2:** **Hemodynamic and biological parameters at the time of sonography**

	**Mean**	**SD**
Mean arterial pressure (mmHg)	91	14
PaCO_2_ (mmHg)	36	4
Serum sodium (mEq/L)	140	5
Body temperature (°C)	36.6	1.2

PaCO_2_, partial pressure of carbon dioxide in arterial blood.

The time between TCS and CT was 83 ± 70 minutes.

### Computed tomography results

The Marshall scores from the patients’ CT scans are presented in the Table 3. Head CTs demonstrated four extradural hematomas (thickness: 1.5 ± 0.75 cm), five subdural hematomas (thickness: 1.2 ± 0.59 cm), nineteen intraparenchymal hematomas (thickness: 4.2 ± 2 cm × 4.4 ± 1.7 cm) as well as thirteen subcutaneous temporal hematomas (thickness: 1.8 ± 1 cm).

**Table 3:** **Marshall score based on CT scan for the study patients**

**Marshall category**	**Number**	**Percentage**
Diffuse Injury I (no visible pathology)	3	5.8%
Diffuse Injury II (cisterns are present with midline shift of 0 to 5 mm and/or lesions densities present; no high or mixed density lesion >25 cm^3^ may include bone fragments and foreign bodies)	4	7.7%
Diffuse injury III (swelling) (cisterns compressed or absent with midline shift of 0 to 5 mm; no high or mixed density lesion >25 cm^3^	13	25%
Diffuse injury IV (shift) (midline shift >5 mm; no high or mixed density lesion >25 cm^3^)	19	36.5%
Evacuated mass lesion (any lesion surgically evacuated)	7	13.5%
Non-evacuated mass lesion (high or mixed density lesion >25 cm^3^; not surgically evacuated)	6	11.5%
Total	52	100%

CT, computed tomography.

MLS was 0.42 ± 0.55 cm (using method 1) and 0.47 ± 0.67 cm (using method 2). A MLS >0.5 cm with CT method 2 was observed in 37% (19/52) of the patients.

### Transcranial sonography results

A MLS measurement was possible in all 52 patients and showed an average MLS of 0.32 ± 0.36 cm. A MLS >0.5 cm was observed in 25% (13/52) of the patients.

**Figure 3:**
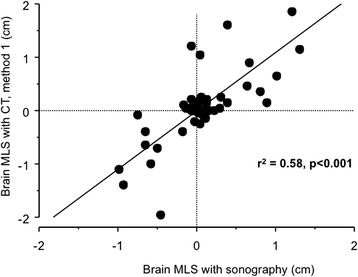
**Correlation between sonography and CT method 1 for MLS assessment.** CT, computed tomography; MLS, midline shift.

**Figure 4:**
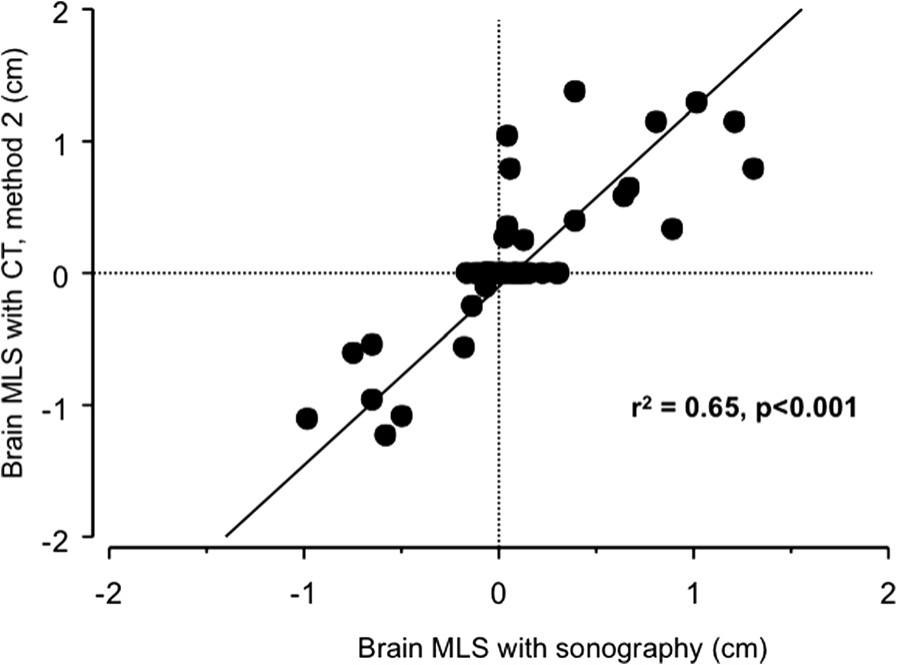
**Correlation between sonography and CT method 2 for MLS assessment.** CT, computed tomography; MLS, midline shift.

**Figure 5:**
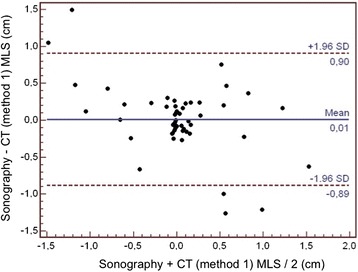
**Bland and Altman plot for the agreement between sonography and CT method 1 for MLS assessment.** CT, computed tomography; MLS, midline shift.

**Figure 6:**
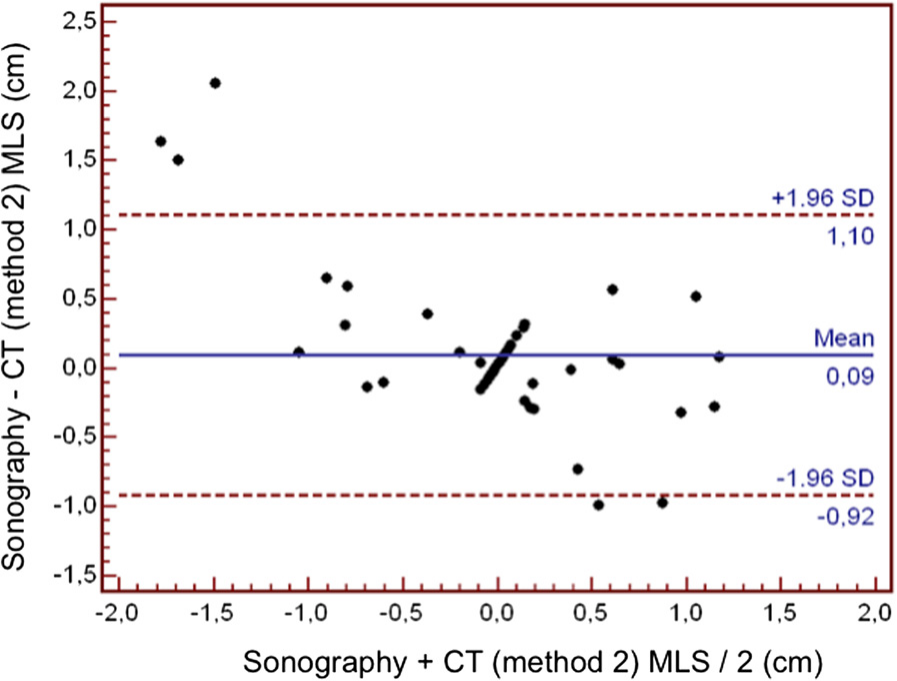
**Bland and Altman plot for the agreement between sonography and CT method 2 for MLS assessment.** CT, computed tomography; MLS, midline shift.

The correlation coefficient (r^2^) between TCS and CT scan was 0.58 with method 1 (*P* <0.001, Figure 3) and 0.65 with method 2 (*P* <0.001, Figure 4). The limits of agreements for MLS measurements with TCS and the two CT methods are presented in the Bland and Altman plots, showing a bias of 0.01 cm and limits of agreement from 0.90 to -0.89 cm for TCS and CT method 1 (with five measures – that is, 9% - outside the limits of agreement; Figure 5) and a bias of 0.09 cm and limits of agreement from 1.10 to -0.92 cm for TCS and CT method 2 (also with five measures – that is, 9% - outside the limits of agreement; Figure 6). As several conditions could have affected the agreement between TCS and CT (method 2), a subgroup analysis was performed for the patients who had undergone a decompressive craniectomy or who had a subcutaneous hematoma. The results are presented in the Table 4.

**Table 4:** **Correlation coefficient, bias and limits of agreement between MLS measurement with sonography versus CT (method 2) in subgroups of patients where the pathology could adversely affect the sonographic measurement**

**Patient characteristics**	**Number**	**r** ^**2**^	**Bias (cm)**	**Limits of agreement (cm)**
Decompressive craniectomy	12	0.85	0.09	-0.66 – 0.48
Intracranial hematoma (SDH, EDH, IPH)	28	0.64	0.09	-1.29 – 1.11
Subcutaneous temporal hematoma	13	0.83	0.14	-1.14 – 0.87
MLS >0.5 cm	19	0.69	0.22	-1.84 – 1.39
Invasive ICP >20 mmHg	9	0.70	0.00	-0.96 – 0.96

CT, computed tomography; EDH, extradural hematoma; ICP, intracranial pressure; IPH, intraparenchymal hematoma; MLS, midline shift; SDH, subdural hematoma.

**Figure 7:**
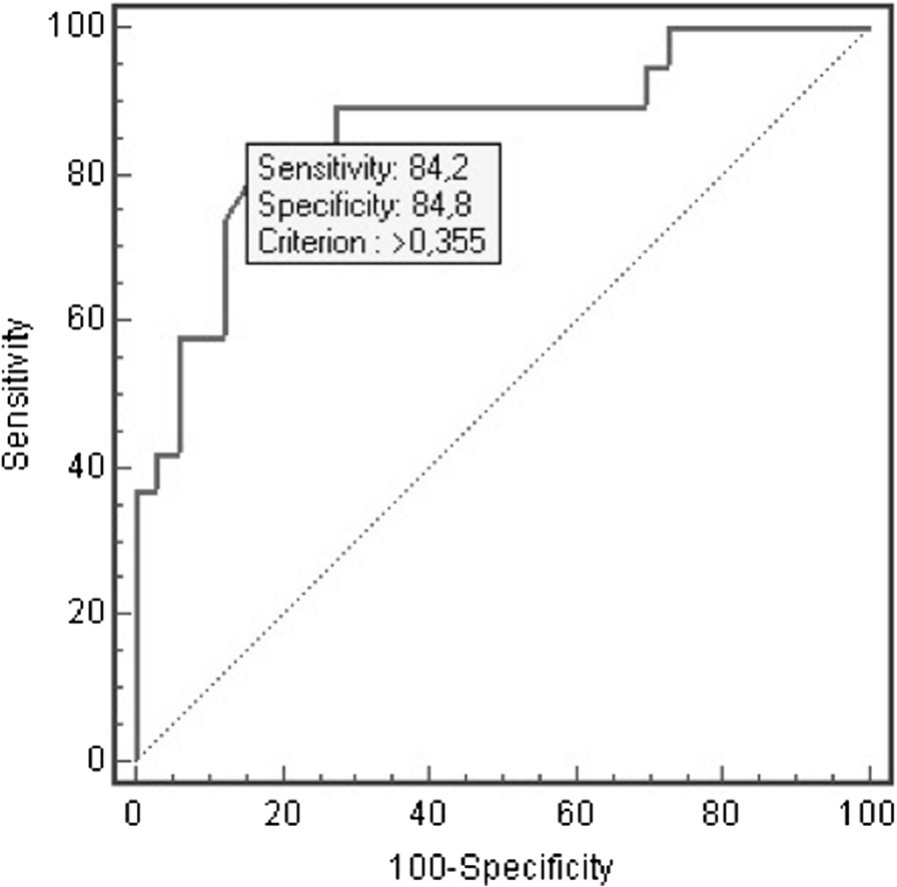
**Receiver operating characteristic curve for the detection of a CT MLS >0.5 cm (method 2) with TCS. CT, computed tomography; MLS, midline shift; TCS, transcranial sonography.**

The sensitivity and the specificity of TCS to detect a significant MLS (that is, MLS >0.5 cm in method 2 of the CT scan) were analysed with the ROC curve. The area under the ROC curve was 0.86 (95% confidence interval (CI) = 0.74 to 0.94%) and, with a cut-off of 0.35 cm, the sensitivity was 84.2% (95% CI = 60.4 to 96.4%), the specificity 84.8% (95% CI = 68.1 to 94.8%) and the positive likelihood ratio 5.56 (Figure 7). When the CT method 1 was used to define a ‘significant’ MLS (that is, MLS >0.5 cm), the area under the ROC curve was 0.85 (95% CI = 0.73 to 0.94%) and with a cut-off of 0.30, the sensitivity was 85.7% (95% CI = 57.2 to 97.8%) and the specificity 84.2% (95%CI = 68.7 to 93.9%).

To study the reliability of the MLS measure with TCS, 20 measures were simultaneously realised with two different sonography units. The bias between both methods was -0.01, and the correlation coefficient was r = 0.72 (*P* <0.0004). The inter-rater weighted kappa was 0.63.

Finally, the relationship between MLS and intracranial pressure was studied by examining the results from the 30 patients with invasive ICP monitoring. The ICP was found to be 19 ± 21 mmHg, with nine patients having an ICP >20 mmHg at the time of TCS. No significant correlation was found between ICP and MLS as assessed with TCS (r = 0.12), CT method 1 (r = 0.14) or CT method 2 (r = 0.06). For the nine patients with raised ICP the bias between CT and TCS MLS was 0.0 cm with limits of agreement from -0.96 to 0.96 (Table 4).

## Discussion

This study suggests that it is possible to detect MLS with a reasonable accuracy in neurosurgical ICU patients with various intracranial pathologies. This could facilitate early diagnosis and treatment for patients with significant intracranial mass effects.

In 1990, Bogdahn *et al*. described for the first time the identification of cerebral structures with sonography and was able to identify the third ventricle in 45 of 52 subjects [22]. Seidel *et al*. subsequently proposed to measure the MLS with ultrasound by setting the centre of the third ventricle as a reference [23]. This method was used to determine the mass effect of patients presenting with acute ischemic stroke and Gerriets *et al.* showed that an ultrasound MLS >0.4 cm within the first 32 hours was associated with a near 100% mortality, with the exception of a patient having undergone decompressive craniectomy [28]. The same team published similar results two years later in 2001 [29].

Among several studies comparing MLS measured by TCS versus CT in neurological [24-27,29] and neurosurgical [30] patients, there was only a small difference between the two measurements (from 0.003 to 0.11 cm) and an acceptable agreement (between 0.18 and 0.35 cm). However, the difference between TCS and CT measurements seems to increase with the size of the MLS, leading to significant underestimation of MLS from TCS in patients with large MLS [25]. One of the limitations in the previous study performed in neurosurgical patients (41 patients with severe TBI) was that 93% of the MLS were less than 0.5 cm and that 8 patients had a decompressive craniectomy and 4 presented with a subcutaneous temporal hematoma [30], making the results of this study difficult to extrapolate to sicker neurosurgical ICU patients (that is, with larger MLS) whereas our study included very ill patients, 37% of whom had a significant MLS on CT (37%) and with raised ICP.

Several methods for estimating MLS using CT have been described, including the measurement of the distance between the ideal mid line and the septum pellucidum [3,32], the horizontal displacement of the pineal gland [15] as well as the difference in the distance from the skull to the centre of the third ventricle between the right and left side [30] (the latter of which has been shown to correlate well with outcome). In our study, the best correlation between TCS and CT was obtained by using the distance on CT between the ideal mid line and the septum pellucidum, with a bias of only 0.09 cm. However, relatively large limits of agreement were observed in the subgroup of patients with a MLS >0.5 cm, where we actually observed a bias of 0.22 cm, probably because of less precision for patients with larger MLS, as previously published by Bertram *et al*. [25]. The correlation coefficient between TCS and CT was slightly better when using CT method 2 compared to method 1 (0.65 versus 0.58). However, the bias was smaller and the limits of agreement were narrower when using CT method 1 (mean bias of 0.01 cm and limits of agreement from 0.90 to -0.89 cm for TCS and CT method 1 versus a mean bias of 0.09 cm and limits of agreement from 1.10 to -0.92 cm for TCS and CT method 2). This could be due to the fact that CT method 1 uses the same imaging plane as TCS, whereas CT method 2 uses a different plane (which is the neuroradiologists’ conventional way to measure MLS). Using a different imaging plane could, thus, have increased the bias between sonography and CT without affecting the correlation.

In our study, we found that TCS seemed to systematically underestimate the CT MLS (the MLS was 0.32 cm with TCS, 0.42 cm with CT method 1 and 0.47 cm with CT method 2). The standard method to measure the MLS with CT was performed at the level of the septum pellucidum, where the deviation was largest and not strictly at the level of the third ventricle [11] and this could explain these findings. In the study of Pullicino *et al*. in 1997, the shift of the septum pellucidum and the pineal gland were highly correlated in acute stroke patients (r = 0.83), but with less deviation for pineal gland, and, therefore, only the deviation of the septum pellucidum was a significant risk factor for 14-day mortality [16]. However, even if MLS may be underestimated by TCS, the ability to detect significant MLS (>0.5 cm in CT scan) with TCS was good in our study, with a sensitivity and specificity around 85% when using a threshold for a significant MLS set at 0.35 cm.

We did not find a correlation between ICP and MLS in our study. This is not surprising. In fact, ICP and MLS have different pathophysiological mechanisms, and the mass effect on the midline does not necessarily indicate raised ICP. To better illustrate this point, a subgroup analysis was performed in the nine patients with raised ICP (that is, >20 mmHg), revealing a bias between CT and TCS MLS of 0.0 cm with limits of agreement from -0.96 to 0.96, a result very close to the one obtained in the whole population.

Our study has several limitations. First, a single operator performed the TCS measurements. While it was, therefore, possible to study the reproducibility of the results comparing different ultrasound devices we could not study inter-observer variability, which could have been of interest to validate this method for clinical use. However, Seidel *et al*. in 1996 had already found a good reproducibility for the measurements in healthy subjects [23]. The reproducibility of the MLS assessment between two different sonography units was tested in the present study, with a good reproducibility as shown by the weighted kappa of 0.63 (a kappa between 0.81 and 0.99 represents an almost perfect agreement, and a kappa between 0.61 and 0.80 represents substantial agreement). Secondly, the measurement of MLS using sonography and CT were not simultaneous with an average delay of 83 ± 70 minutes and, as intracranial mass effects can change rapidly, it is possible that the decrease in agreement between methods could in part be due to each method measuring a changing MLS at different time points.

Our study adds to the existing knowledge, showing that a MLS >0.35 cm on TCS in severely ill neurosurgical ICU patients, including patients after decompressive craniectomy or with subcutaneous temporal hematomas, can predict a MLS >0.5 on CT with good sensitivity, specificity and a positive likelihood ratio of more than 5. Given that this cut-off on CT is recognized as a major prognostic factor and generally accepted to constitute an urgent indication for a neurosurgical intervention, MLS assessment with TCS could potentially be considered as an alternative diagnostic method to serial CTs for selected patients. Furthermore, our study is the first to compare MLS measurement using TCS to measuring it via the displacement of septum pellucidum on CT, which is the most frequently used method for MLS assessment with CT [11]. Our data also revealed that MLS measurements are reproducible across two different ultrasound units. Finally, whereas in all previously published studies trained or expert operators performed sonography, in the present study the operator did not have extensive experience in brain sonography and the accuracy of TCS in determining MLS could theoretically be even higher in experienced hands.

## Conclusions

This study suggests that TCS could detect MLS with reasonable accuracy in neurosurgical ICU patients and that it could serve as a bedside tool to facilitate early diagnosis and treatment for patients with a significant intracranial mass effect.

## Key message

- Brain midline shift can be accurately estimated using brain sonography.

## Abbreviations

CI: confidence interval
CT: computed tomography
GCS: Glasgow Coma Scale
ICP: intracranial pressure
ICU: intensive care unit
MLS: midline shift
ROC: receiver operating characteristic
SAPS II: Simplified Acute Physiologic Score
TBI: traumatic brain injury
TCS: transcranial sonography
